# Choroidal vascularity features of fundus tessellation in adults with high myopia

**DOI:** 10.1186/s12886-024-03567-7

**Published:** 2024-07-22

**Authors:** Jiarui Xue, Rongrong Zhang, Minmin Zheng, Xiao Cao, Chenhao Li, Changfan Wu

**Affiliations:** 1 Department of Ophthalmology, Yijishan Hospital of Wannan Medical College, 92 West Zheshan Road, Wuhu, Anhui Province 241001 China; 2 Department of Ophthalmology, Fuyang People’s Hospital Affiliated to Anhui Medical University, Fuyang, Anhui Province 236000 China

**Keywords:** High myopia, Fundus tessellation, Choroidal vascularity, Factors, Binarization, EDI-OCT

## Abstract

**Background:**

To investigate alterations in choroidal vascularity index among highly myopic adults with fundus tessellation, utilizing optical coherence tomography.

**Methods:**

Total of 143 highly myopic adults (234 eyes) with fundus tessellation were collected in this cross-sectional study, which was stratified into different lesion groups based on the novel tessellated fundus classification. Subfoveal choroidal thickness (SFCT), choroidal luminal area (LA), stromal area (SA), total choroidal area (TCA), and choroidal vascularity index (CVI) were analyzed utilizing optical coherence tomography (OCT) with enhanced depth imaging (EDI) mode, enabling precise quantification of these parameters.

**Results:**

Comparison analysis demonstrated notable distinctions in spherical equivalent (SE), axial length (AL), and SFCT across the four tessellation grades (*p* < 0.001). Analysis of the choroidal vascularity parameters, including LA, TCA, and CVI, demonstrated notable disparities across the four groups (*p* < 0.001), while no significant variations were observed in SA when comparing Grade 1 versus Grade 2, as well as Grade 2 versus Grade 3 (*p* > 0.05). Logistic regression analyses illustrated that the higher grade of tessellated exhibited a positive association with AL (OR = 1.701, *p* = 0.027), while negatively associated with SFCT (OR = 0.416, *p* = 0.007), LA (OR = 0.438, *p* = 0.010) and CVI (OR = 0.529, *p* = 0.004). Multiple regression analyses demonstrated a significant negative association between CVI and both SE and AL after adjusting for age, while positively associated with SFCT (*p* < 0.05).

**Conclusion:**

Subtle choroidal vascularity changes may have a meaningful contribution to the development and progression of fundus tessellation. CVI and LA dramatically decreased during the early stages of tessellation development and maintained a relatively stable status when in the severe tessellated grades.

## Background

Myopia represents a significant challenge to global public health, with projected increases in its prevalence from an estimated 33.9% of the population in 2020 to a predicted 49.8% by 2050. Additionally, the occurrence of high myopia - a condition frequently associated with vision impairment - is anticipated to rise from 5.2 to 9.8% globally, with a particularly sharp increase anticipated in East Asia [1–4]. Notably, high myopia is frequently linked to an elongated axial length which may give rise to several serious and irreversible ocular conditions such as myopic maculopathy, retinal detachment, myopic optic neuropathy, and glaucoma, all of which pose substantial risks to visual function [5, 6]. Given the higher prevalence and severity of high myopia and myopia-related complications, the study of earlier-stage fundus lesion characteristics is warranted.

Fundus tessellation, the initial manifestation of myopic maculopathy, is characterized by the visualization of plainly delineated choroidal vessels around the fovea and arcade vessels according to the META Classification System [7]. Several subsequent investigations have consistently shown that the early tessellated fundus is typically identified in individuals with the following traits when contrasted with those exhibiting more severe ocular pathologies including younger age, good best-corrected visual acuity, lower degree of myopia, shorter axial length, and reduced occurrence of staphyloma. [8–10]. Additionally, previous research has revealed that tessellated fundus stage can be stable for a long time without progression, whereas the choroidal thickness (CT) significantly decreases from tessellation to peripapillary diffuse choroidal atrophy (PDCA), with an optimal value of 56.5 μm in the nasal region from the fovea, which could be utilized as a quantitative index to evaluate myopia severity progression [9, 11]. As the choroid is predominantly a vascular structure, alterations in the choroidal thickness may stem from modifications in the choroidal vasculature, thereby contributing to the progression of myopia [12]. It should be acknowledged that the CT, as a standalone parameter, may not offer a full and accurate reflection of the entire choroidal vasculature, being extensively influenced by many physiological and ocular variables, which may lead to possible confounders and an incomplete analysis [13].

The evolution of optical coherence tomography (OCT) technology has introduced EDI-OCT, offering superior resolution and deeper imaging capabilities. This advancement enables detailed visualization and a more in-depth analysis using novel quantitative imaging biomarkers, enhancing the assessment of the choroid’s influence on various ocular conditions. [14]. The choroidal vascularity index (CVI), which was defined as the ratio of the choroidal luminal area to the total choroidal area, has shown promise as a noninvasive index for early diagnosis, disease progression monitoring and prognosis evaluation of choroidal microcirculation conditions [13]. Previous investigations have shown that the CVI may serve as a robust marker in myopia and various fundus diseases including diabetic retinopathy [15], central serous chorioretinopathy [16], panuveitis [17], age-related macular degeneration [18, 19], and retinitis pigmentosa [20]. Wu et al. recently also reported that the CVI was negatively correlated with the AL and severity of myopia [21]. While investigating the choroidal structure changes in tessellated fundus, the majority of research has been predominantly limited to the changes in CT, the thickness of the vascular layer, and the overall volume of the choroid. [22–24]. Lyu and colleagues proposed a new method for grading fundus tessellation that relies on the ETDRS grid in fundus photographs and reported an inverse relationship between the CT and fundus tessellation grade [24]. The primary goal of this study was to utilize the novel grading system to quantitatively evaluate choroidal vascularity characteristics and their potential connections to fundus tessellation in adults with high myopia.

## Materials and methods

### Study population

In this cross-sectional study, we recruited a cohort of 143 highly myopic individuals (234 eyes) with fundus tessellation who underwent both fundus photography and EDI-OCT examinations at our hospital, including 60 males (95 eyes) and 83 females (139 eyes). The inclusion criteria included adults (aged 18 years or older), with an intraocular pressure (IOP) ranging from 10 to 21 mmHg, a refractive error ≥ − 6.0 D or an axial length ≥ 26.0 mm, and a fundus tessellation grade equal to or exceeding to Grade 1 according to the new classification [24]. The exclusion criteria were other myopic complications (including lacquer cracks, choroidal neovascularization, and macular traction syndrome); a previous diagnosis of an ocular or systemic disease, including but not limited to congenital cataract, glaucoma, hypertension, and diabetes; the presence of visual field defects; a history of intraocular or refractive surgeries; and images that exhibited a poorly defined the choroidal–scleral interface (CSI). The present study followed the ethical principles of the Declaration of Helsinki and was granted approval by the Ethics Committee of the Yijishan Hospital prior to commencement. (2019A052).

### Classification criteria

To classify the tessellation lesions, we utilized a recently developed grading system that incorporates an ETDRS grid to determine the position of lesions with respect to the fovea [24]. This new proposed classification divides the lesions into five distinct grades including Grade 0, no large choroidal vessels visible; Grade 1, fundus tessellations visible in the posterior pole without involving ETDRS grid; Grade 2, fundus tessellations visible in the outer circle of ETDRS grid without involving the near circle; Grade 3, fundus tessellations visible in the middle circle of ETDRS grid without involving the inner circle; and Grade 4, fundus tessellations visible in the center fovea of ETDRS grid. The photographs included were read by two qualified ophthalmologists (XC and CL) in a concealed analysis to determine the degree of tessellation. A senior ophthalmologist (CW) was involved in the decision-making process when disagreement occurs regarding the grade.

### Ocular biometric measurements

A thorough ophthalmologic assessment was conducted on all participants and involved anterior segment evaluation using slit-lamp biomicroscopy, measurement of IOP with a noncontact tonometer (NT-510, Japan), spherical equivalent refraction measurements with an autorefractometer without the use of cycloplegic agents and a calculation by adding the sphere value to half of the cylindrical refraction (KR-8900, Topcon, Tokyo, Japan), and recording the axial length with an IOL Master (Carl Zeiss Meditec, Jena, Germany). TRC-50DX was utilized to acquire digital color fundus photographs, which were centred on the macula within a 45°field (Topcon, Tokyo, Japan). Spectral domain OCT in EDI mode was employed to capture horizontal scans traversing the fovea, and raster scans that encompassed all macular abnormalities were acquired (Heidelberg Engineering, Heidelberg, Germany). The subfoveal choroidal thickness (SFCT) was manually measured by using the calliper tool on the OCT device, which involved determining the vertical distance between the Bruch membrane and the CSI as it passed through the centre of the fovea.

### Choroidal vascularity acquisition

**Fig. 1:**
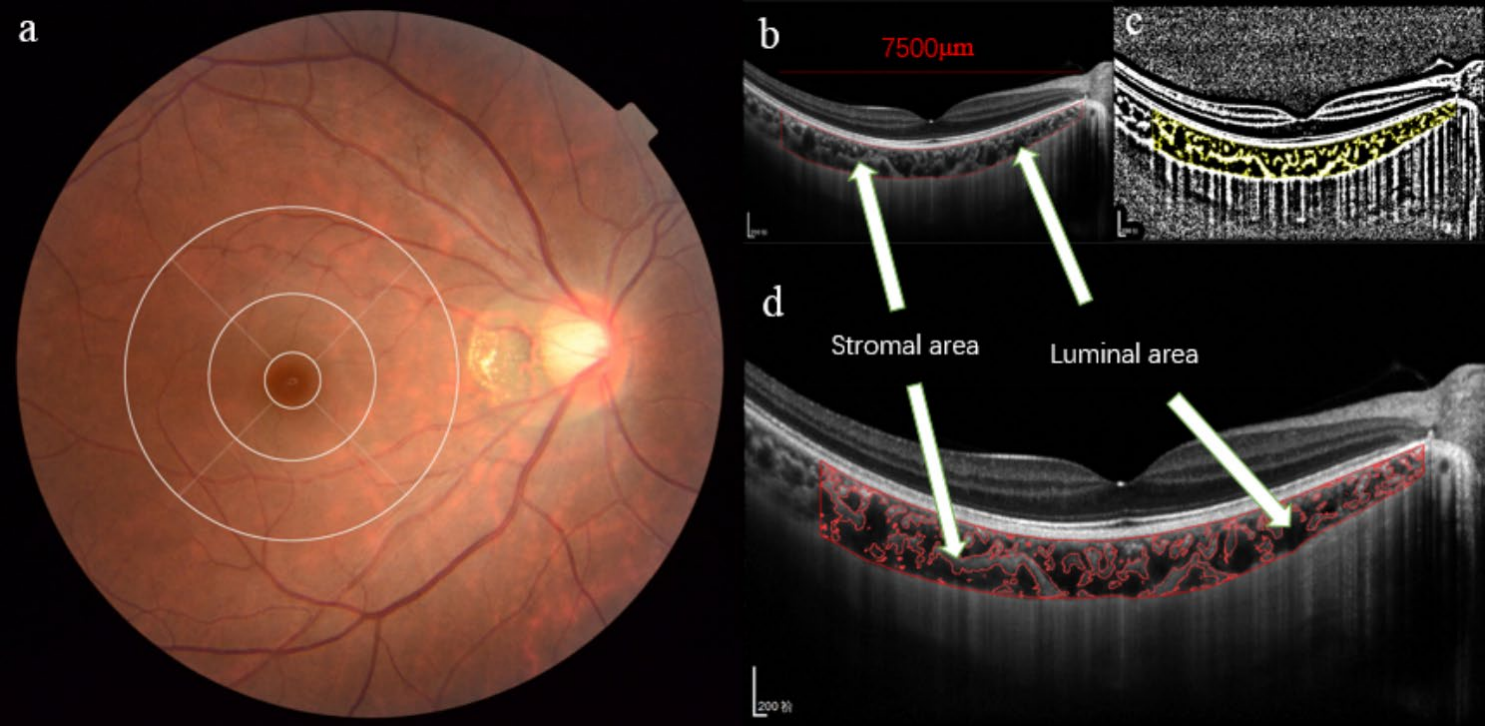
The application of ETDRS grid in fundus tessellation grading and binarization analysis of choroidal structure from correspondent OCT images. (**a**) Right eyes with fundus tessellation involving the middle circle were graded as Grade 3. (**b**) Determination of the choroidal region (7500 μm central for foveal) in the original EDI-OCT image with the ImageJ ROI manager. (**c**) Identification of the choroidal regions utilizing the binarization technique. (**d**) Overlay of the target choroidal region established by image binarization on the EDI-OCT image

Binarization of the subfoveal choroidal area was carried out using a refined Niblack method on a horizontal line scan with a width of 7500 μm (3750 μm on either side of the fovea) from the EDI-OCT images; this process was performed using the ImageJ software version 1.53e (available at: http://imagej.nih.gov/ij/). The polygon tool was utilized to specify the area of the region of interest (ROI). The vertical expansion of the total choroidal area (TCA) from the retinal pigment epithelium (RPE) as the upper border to the CSI as the lower border was determined manually using the ROI manager. Furthermore, the image was subjected to the conversion to 8 bits and adjusted using an autolocal threshold (Niblack method), which facilitated the differentiation between the luminal area (LA) and the stromal area (SA). Upon binarization, the image was transformed back to an RGB format, and the color threshold tool was employed to identify the LA. The ROI manager allowed for the selection and subsequent merging of both areas of interest, resulting in a consolidated ROI. The CVI index was defined as the ratio of the choroidal LA to the TCA. Figure 1 shows examples of tessellated fundus photographs and corresponding images binarized with EDI-OCT, both in the original form and after the conversion process.

### Statistical analysis

SPSS version 26.0 was utilized for conducting the statistical analysis (Chicago, IL, USA). Continuous variables were represented as the mean ± standard deviation, while discrete variables were described as counts (proportions). To evaluate the differences in demographic and ocular parameters between the four groups, the Kruskal-Wallis H test was performed for variables with non-normal distribution, while the one-way analysis of variance (ANOVA) test was used for normally distributed variables. To assess categorical data, the Chi-square test was employed. Multiple logistic regression analysis was conducted to explore the relationship between the grade of fundus tessellation and ocular or systemic parameters, and the linear regression analysis was carried out to determine the influence of other variables on the CVI. Statistical significance was considered as p value less than 0.05.

## Results

### Demographic and ocular characteristics

A total of 143 highly myopic adults were included in this study, and fundus color photographs and OCT images were obtained from 234 eyes for the final analysis via the ETDRS grid method to further investigate changes in choroidal vascularity. The general characteristics of the 143 adults with tessellation were described as follows. The mean age of subjects enrolled was 42.63 ± 14.49 years (range: 18 to 75 years), and 83 (58.04%) of the participants were females. The average values of the IOP, SE and AL were 14.32 ± 2.43 mmHg, -11.05 ± 3.82 D (range: -6.00 D to -22.75 D) and 27.45 ± 1.21 mm (range: 26.0 to 30.79 mm), respectively. Considering the choroid parameters, the mean SFCT value was 183.71 ± 65.21 μm (range: 70 to 317 μm), and the average values of the LA, SA, TCA and CVI were 0.85 ± 0.36 mm^2^, 0.72 ± 0.09 mm^2^, 1.57 ± 0.42 mm^2^, and 52.0 ± 8.42%, respectively.

### Characteristics of different tessellation grades

**Fig. 2:**
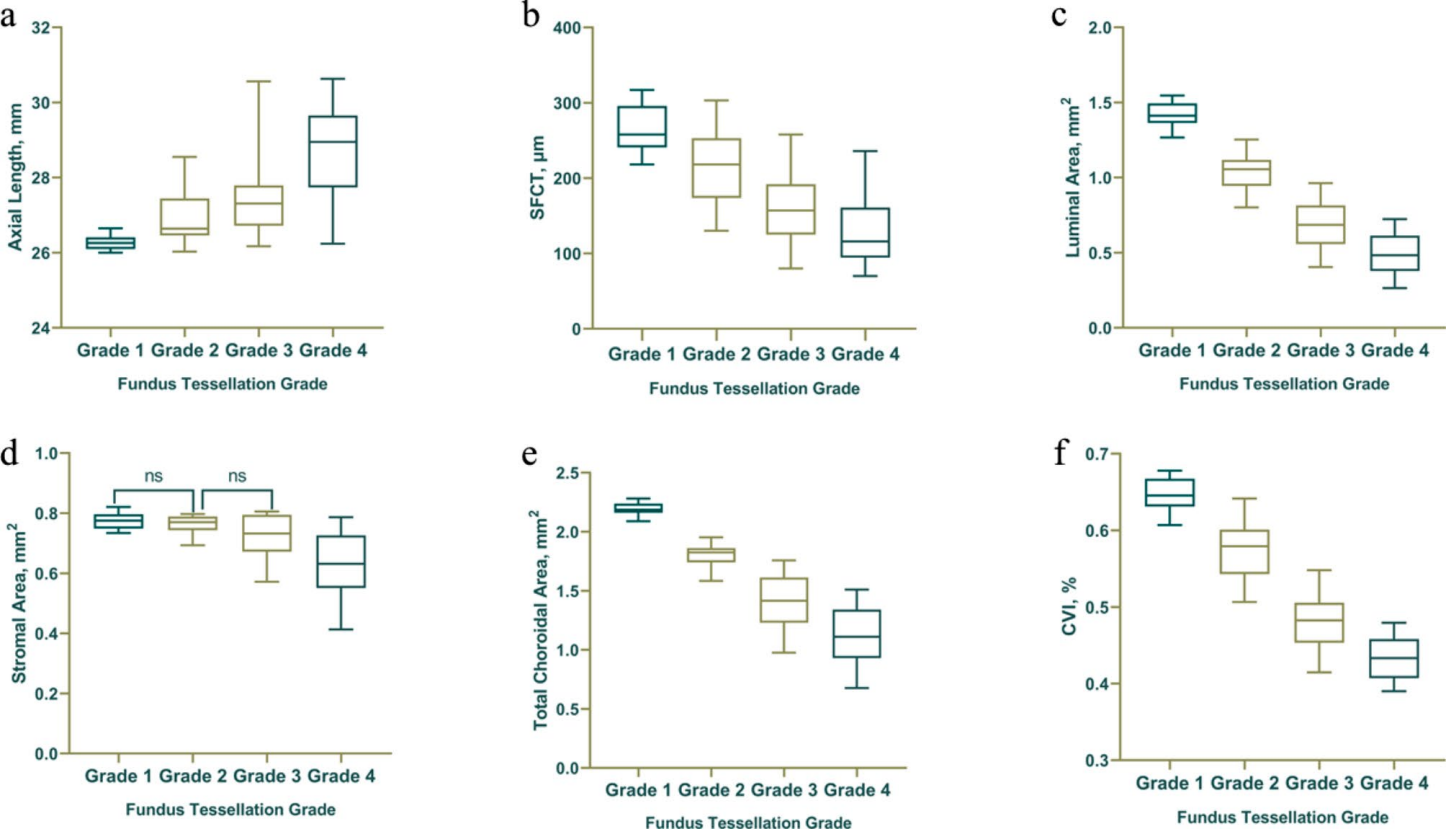
Box plots demonstrating the changes in choroidal parameters with increasing grader. **a** change in axial length, **b** change in subfoveal choroidal thickness, **c** change in LA, **d** change in SA, **e** change in TCA and **f** change in CVI with increasing grader. Ocular parameters showed significant differences among the four groups (*p* < 0.001), while no significant difference was found in the comparison of SA between Grades 1 and 2, and Grades 2 and 3 (*p* > 0.05)

Fundus tessellation of Grade 1, 2, 3 and 4 in the posterior pole occurred in 42 (17.95%), 57 (24.36%), 71 (30.34%) and 64 (27.35%) eyes, respectively. Table 1 presents a comparison of systematic and ophthalmologic parameters across different grades of fundus tessellation. No significant differences were observed in terms of sex or IOP among the four groups. The parameters of age, SE, AL and SFCT showed significant differences across the different grades of tessellation (*p* < 0.001). Significant variations were evident in the choroidal vascularity parameters, such as the LA, TCA and CVI, across the four groups (*p* < 0.001). In addition, as displayed in the Fig. 2, the SA was significantly different among the four groups (*p* < 0.01), except for the comparisons between Grade 1 and Grade 2 and between Grade 2 and Grade 3 (*p* > 0.05).

**Table 1:** Comparative evaluation of demographic and ocular variables across distinct grades of tessellated fundus

Variables	Grade 1	Grade 2	Grade 3	Grade 4	*p*
No. of eyes	42	57	71	64	-
Age, years	38.83 ± 12.46	39.86 ± 13.80	41.17 ± 14.51	49.22 ± 14.56	< 0.001
Female, n (%)	27 (64.29)	38 (66.67)	39 (54.93)	35 (54.69)	0.419
IOP, mmHg	14.35 ± 2.37	14.32 ± 2.46	14.46 ± 2.58	14.16 ± 2.30	0.995
SE, D	-7.18 ± 0.97	-9.63 ± 2.20	-11.47 ± 2.98	-14.40 ± 3.95	< 0.001
AL, mm	26.27 ± 0.18	26.90 ± 0.60	27.47 ± 0.89	28.69 ± 1.17	< 0.001
SFCT, µm	265.83 ± 30.28	214.23 ± 46.35	159.32 ± 45.11	129.69 ± 44.53	< 0.001
LA, mm^2^	1.420 ± 0.08	1.036 ± 0.12	0.686 ± 0.15	0.491 ± 0.14	< 0.001
SA, mm^2^	0.773 ± 0.03	0.765 ± 0.03	0.727 ± 0.07	0.629 ± 0.11	< 0.001
TCA, mm^2^	2.193 ± 0.05	1.801 ± 0.09	1.412 ± 0.21	1.120 ± 0.25	< 0.001
CVI, %	64.72 ± 2.07	57.35 ± 3.56	48.09 ± 3.18	43.23 ± 2.75	< 0.001

SD: Standard deviation; IOP: Intraocular pressure; SE: Spheric equivalent; D: Diopter; AL: Axial length; SFCT: Subfoveal choroidal thickness; LA: Luminal area; SA: Stromal area; TCA: Total choroidal area; CVI: Choroidal vascularity index

Multivariable logistic regression models were performed to identify the factors closely linked to the fundus tessellation grade, and the results are shown in Table 2. A greater degree of fundus tessellation was positively correlated with the AL (OR = 1.701, 95% CI: 0.740–1.928, *p* = 0.027) but negatively correlated with the CVI (OR = 0.529, 95% CI: 0.426–0.658, *p* = 0.004), LA (OR = 0.438, 95% CI: 0.233–0.823, *p* = 0.010) and SFCT (OR = 0.416, 95% CI: 0.220–0.784, *p* = 0.007).

**Table 2:** Multivariable logistic regression model analysis for tessellated fundus grades

Variables	Model 1	Model 2	Model 3	Model 4	Model 5	Model 6	Model 7
	OR	OR	OR	OR	OR	OR	OR
Age, years	1.003	1.212	1.184	0.975	0.879	1.134	1.011
SE, D	-	1.343^*^	1.267^*^	1.042	1.175	1.207	1.252
AL, mm	1.662^*^	-	1.731^*^	1.536^*^	1.632^*^	1.672^*^	1.701^*^
SFCT, µm	0.526^*^	0.422^*^	0.444^*^	0.667^*^	0.414^*^	0.433^*^	0.416^*^
LA, mm^2^	0.455^*^	0.415^*^	-	0.584	0.538^*^	0.480^*^	0.438^*^
SA, mm^2^	1.144	1.538	1.633^*^	-	1.286	1.404	1.478
TCA, mm^2^	1.079^*^	1.107	1.232	1.341^*^	-	1.311	1.201
CVI, %	0.593^*^	0.469^*^	0.604^*^	0.575^*^	0.622^*^	-	0.529^*^

SE: Spheric equivalent; D: Diopter; AL: Axial length; SFCT: Subfoveal choroidal thickness; LA: Luminal area; SA: Stromal area; TCA: Total choroidal area; CVI: Choroidal vascularity index; OR: Odds ratio

Model 1: Adjusted for SE

Model 2: Adjusted for AL

Model 3: Adjusted for LA

Model 4: Adjusted for SA

Model 5: Adjusted for TCA

Model 6: Adjusted for CVI

* *P* < 0.05

### Choroidal vascularity index

Further analyses were carried out using both univariable and multivariable linear regression to determine the variables linked to the CVI, and the results were shown in Tables 3 and 4. The results demonstrated significant inverse correlations between the CVI and age, SE, and AL (*p* < 0.001), while the age distribution was significantly different only in the Grade 4 tessellation group. Multiple regression analyses demonstrated significant negative associations between the CVI and both SE and AL with age as a covariate (*p* < 0.05) but a significant positive association with the SFCT (*p* < 0.05).

**Table 3:** Linear regression analysis the potential correlation between systemic and ocular factors and CVI

	Univariable					Multivariable				
	Unstandardized β		Standardized β		p	Unstandardized β		Standardized β		p
Age		-0.156		-0.269	< 0.001	-0.004	-0.006		0.860	
Sex		-0.215		-0.316	0.223	-	-		-	
IOP		0.010		0.003	0.965	-	-		-	
SE		-0.586		-0.707	< 0.001	-0.445	-0.569		< 0.001	
AL		-0.626		-0.843	< 0.001	-0.636	-0.706		< 0.001	
SFCT		0.815		0.857	< 0.001	0.885	0.838		< 0.001	

IOP: Intraocular pressure; SE: Spheric equivalent; AL: Axial length; SFCT: Subfoveal choroidal thickness; β: Regression coefficient

**Table 4:** Linear regression analysis the potential correlation between systemic and ocular factors and CVI according to different tessellated grades

	Grade 1	Grade 2	Grade 3	Grade 4		Grade 1	Grade 2	Grade 3	Grade 4
Variables	Univariable					Multivariable			
	Standardized β					Standardized β			
Age	-0.113	-0.009	-0.182	-0.294^*^		-	-	-	-0.013
SE	-0.377^*^	-0.782^*^	-0.775^*^	-0.681^*^		-0.214	-0.577^*^	-0.543^*^	-0.441^*^
AL	-0.782^*^	-0.887^*^	-0.845^*^	-0.823^*^		-0.533^*^	-0.781^*^	-0.696^*^	-0.734^*^
SFCT	0.814^*^	0.887^*^	0.863^*^	0.832^*^		0.755^*^	0.826^*^	0.883^*^	0.874^*^

SE: Spheric equivalent; AL: Axial length; SFCT: Subfoveal choroidal thickness; β: Regression coefficient

*Statistically significant difference

## Discussion

The establishment of a new classification system for fundus tessellation with an ETDRS grid provides a relatively detailed distinction, which facilitates the effective comparison and analysis of choroidal structures in highly myopic adults. Including a SD-OCT examination with EDI mode, along with the binarization method for measuring choroidal vascularity, may provide a higher- resolution visualization and quantification of choroidal components.

Earlier investigations revealed a noteworthy correlation between the fundus tessellation grade and choroidal components in both children and adults [8, 25, 26], which is consistent with our findings showing that the tessellation grade was correlated with the AL and SFCT. According to the Beijing Eye Study, higher tessellation grades were correlated with a thinner SFCT and a longer AL among the older population [8]. In a school-based study, Guo et al. [25] reported that the SFCT and CT at a distance of 3-mm to the nasal and temporal sides of the fovea, as well as the AL, were correlated with the tessellation grade. Additionally, Yoshihara et al. [26], who converted the RGB (red, green, and blue) pixels of fundus images into three tessellated fundus indices (TFIs) to objectively classify fundus tessellation, also established a relationship between the CT and these TFIs in the area extending from the fovea to the optic disc.

In several of these studies, the CT was predominantly considered a surrogate indicator for evaluating structural changes in the choroid. Fang et al. [11], based on the OCT examination, provided a threshold value of 56.5 μm nasal to the fovea for the CT that could predict the presence of PDCA from tessellation. However, the CT is representative of only the overall structural changes in the choroid and may not be a reliable indicator of alterations occurring in the choroidal subcomponents throughout disease progression; the CT is also frequently influenced by many physiological and ocular variables, including age, AL, IOP, luminal area, and systolic blood pressure [27]. The CVI is a novel EDI-OCT based metric and a robust marker that provides a precise insight into the choroidal vascular architecture through the use of a binarization method [13]. Previous studies have shown the robust characteristics of the CVI both in healthy individuals and in individuals with various diseases affecting the choroid [15–18, 20, 28]. In the context of this investigation, we utilized the CVI to evaluate choroidal vascularity changes and found that the CVI and LA decreased with the increasing fundus tessellation severity.

Earlier studies have investigated changes in choroidal vascularity parameters in highly myopic children or adults [28, 29]. An initial study by Gupta et al. [29] revealed a decrease in both the choroidal LA and SA, as well as a decrease in the CT, among individuals with high myopia, while the CVI increased, in contrast to that in healthy eyes. Recently, Li and colleagues investigated the effects of low to moderate myopia in children and demonstrated that the LA decreased with an increasing AL and that the SA tended to decrease with an increasing age, whereas the CVI was not correlated with the AL and was only associated with the age [28]. Different from the above findings, our results showed that the severity of tessellation was associated with reductions in both vascular and stromal components. The variations in the outcomes could be ascribed to differences in the OCT equipment, scan methodologies, age of participants and ethnic diversity. The present study revealed that the choroidal LA, SA, TCA and CVI decreased with an increasing tessellation grade, and the vascular lumen area exhibited a greater reduction than did the stromal area. The comparative analysis of the groups revealed a slight decrease in the choroidal SA between Grade 1 and Grade 2, as well as between Grade 2 and Grade 3. In contrast, the choroidal LA significantly decreased, particularly at the lower grade levels.

Progression of fundus tessellation from Grade 1 to Grade 2 and further to Grade 3 with a significant decrease of choroidal LA than SA, leading to a substantial decrease of CVI, while the vascularity parameters were the only modest changes from Grade 3 to Grade 4. We speculated that with the greater decrease of choroidal LA, which may be attributed to impaired vasodilatation caused by blood vessels dysfunction, with a slight worsening of choroidal SA results from a persistent proinflammatory response to culminate fibrosis, these factors together would contribute towards the reduced CVI. And these choroidal vascularity parameters dramatically decreased during the early tessellated development and maintained stable status when progressed into severity level. We suggest that these results may be due to the compensation of peripheral choroid vascularity as a result of temporary improvements in blood supply brought about by the nonvascular smooth muscle when fundus lesions intervene in the macula. Therefore, further studies on choroidal vascularity changes during tessellated fundus development should be performed.

Linear regression analyses showed that the CVI was significantly linked to age, SE, AL and SFCT (*p* < 0.001), while the age distribution was significantly different only in Grade 4 patients. These findings were partially different from those of earlier studies. Thus, Wu et al. demonstrated an inverse relationship between the CVI and AL, with prominent changes manifesting in the temporal and inferior macular regions [21]. According to the study conducted by Li et al. [28], the CVI had no meaningful relationship with the AL and was only associated with the age in myopic children. Koçak et al. [30] investigated choroidal structures in healthy populations and found that the decreased LA, TCA, and CVI were related to an advancing age, and a decreased SFCT was significantly associated with the reductions in the LA and SA. These differences mainly arise from differences in the participants’ refraction status, age distributions and different group divisions, as Ruiz-Medrano et al. reported significant differences in the choroidal cross-sectional area, vascular area, and CVI between adult and pediatric population [31]. Previous research has suggested that scleral hypoxia can result in chorioretinal hypoperfusion in myopic eyes [32], while vascular compromise and vascular hypoperfusion may occur as a result of the extending and thinning of both the retina and choroid [33]. The decrease in the CVI, which represents the ratio of the luminal area to the total choroidal area (comprising both the lumina and stroma), suggested that the absence of the choroidal vascular component may have a greater detrimental effect on myopia development than the absence of the stroma. Our results indicated that the mechanical strain resulting from axial elongation could cause a significant reduction in the CVI, which may have important implications for myopia progression. Hence, a long-term observational study exploring the fluctuations in choroidal vascularity components across different age spans in different grades of tessellation is warranted.

Previous investigations have suggested that variances in the scanned area could potentially influence the findings [30, 34]. Thus, in a recent study, Koçak and colleagues reported that the CVI was greater in the 1500-µm subfoveal area than in the overall choroidal region [30]. Kakiuchi et al. [34] also concluded that the CVI significantly increased in the macular region compared with that in the paramacular zone. Sonoda et al. determined that selecting a wider region of the single horizontal line scan, spanning 7500-µm, was more appropriate for accurately determining the binarization of the choroidal area, which was due to the circumvention of increased variation in the luminal/stromal area ratio when using the smaller sampling areas [35]. These factors only relatively influenced the results because of the different areas included. The evaluation of the CVI, in this study, necessitated the binarization of a single cross-sectional scan that was horizontal in orientation and spanned 7500 μm across the fovea. In recent articles, the three-dimensional CVI was analysed in myopic eyes using the ratio of the choroidal vessel volume (CVV) to the entire choroidal volume, and it was found that the CVI, CVV and CT exhibited a predominantly negative associations with the AL [36, 37]. Three-dimensional CVV and CVI, when applied to encompass a wide region and cover a broader range of the AL, may be more effective and informative in demonstrating myopia-related changes in choroidal blood perfusion. We will subsequently apply the three-dimensional CVV and CVI obtained from SS-OCT/SS-OCTA to validate the results and compensate for the limitations of this study.

This study has several limitations that should be considered. First, the CVI was obtained via two-dimensional examination mode. Second, the new method for assessing fundus tessellation in this study relied exclusively on color fundus photographs with the ETDRS grid, which presented some subjective bias. However, the evaluators were highly skilled ophthalmologists with full experience in this particular field. Third, the CT and choroidal vascular parameter (LA, SA and CVI) measurements were taken manually, which could cause differences in the results compared with the findings of other studies. Finally, the agreement between the two qualified ophthalmologists was not determined. Conversely, this study’s design had the advantages of a relatively substantial sample size, the performance of the new classification and the application of the binarization method in CVI measurements.

## Conclusion

In conclusion, this study applied the ETDRS grid classification and the binarization method of CVI measurement on investigating the choroidal vascularity changes in tessellated fundus. Both SFCT and vascularity parameters were reduced with tessellated fundus grades in highly myopic adults. These choroidal vascularity parameters dramatically decreased during the early tessellated development and retained stable status when progressed into severity level, which may be helpful for the differential and diagnosis of severe tessellation lesions and can provide a preventive warrant. The tessellated fundus stage, which represents the initial phase of myopic maculopathy, may remain stable for an extended period. So, analyzing the characteristic changes of tessellated fundus at different degrees of high myopia is conducive to further obtaining indicators of early choroidal vascularity changes to control the accelerated development of this stable period, implement prompt intervention and preventive measures.

## Data Availability

The data underpinning the results of this study can be made available by the corresponding author upon reasonable request.

## Abbreviations

CVI: Choroidal Vascularity Index
SFCT: Subfoveal Choroidal Thickness
LA: Luminal Area
SA: Stromal Area
TCA: Total Choroidal Area
OCT: Optical Coherence Tomography
EDI: Enhanced Depth Imaging
SE: Spherical Equivalent
AL: Axial Length
CT: Choroidal Thickness
PDCA: Peripapillary Diffuse Choroidal Atrophy
IOP: Intraocular Pressure
CSI: Choroidal–Scleral Interface
ROI: Region of Interest
RPE: Retinal Pigment Epithelium
OR: Odds Ratio
CI: Confidence Interval
TFIs: Tessellated Fundus Indices
